# Heterogeneity in Mitogen-Activated Protein Kinase (MAPK) Pathway Activation in Uveal Melanoma With Somatic *GNAQ* and *GNA11* Mutations

**DOI:** 10.1167/iovs.18-26452

**Published:** 2019-06

**Authors:** Getachew Boru, Colleen M. Cebulla, Klarke M. Sample, James B. Massengill, Frederick H. Davidorf, Mohamed H. Abdel-Rahman

**Affiliations:** 1 Department of Ophthalmology, the Ohio State University, Columbus, Ohio, Unites States; 2 Division of Human Genetics, the Ohio State University, Columbus, Ohio, United States

**Keywords:** uveal melanoma, GNAQ, Targeted therapy, GNA11, MAP Kinase

## Abstract

**Purpose:**

The activation of the mitogen-activated protein kinase (MAPK) pathway has been suggested as the major downstream target when *GNAQ* and *GNA11* (*GNAQ/11*) are mutated in uveal melanoma (UM). However, clinical trials with single agent MEK inhibitor showed no clinical significance in altering the overall outcome of the disease in UM; therefore, we investigated the correlation between naturally occurring mutations in *GNAQ/11* and activation of MAPK pathway in vivo in primary UM.

**Methods:**

Screening for activating mutations in codons 183 and 209 of *GNAQ/11* was carried out by sequencing and restriction fragment length polymorphism (RFLP) in a cohort of 42 primary UM. Activation of the MAPK pathway and other potential downstream signals was assessed by immunohistochemistry and/or Western blot analysis. Potential downstream signaling of mutant and wild type *GNAQ/11* was studied by transient transfection assay in nonmutant cell lines.

**Results:**

Somatic mutations in *GNAQ/11* were observed in 35/42 (83.3%) of primary UM. Tumors with *GNAQ/11* mutations showed variations in the activation of ERK1/2 with significant tumor heterogeneity. Weak and undetectable ERK1/2 activation was observed in 4/35 (11.4%) and 8/35 (22.9%) of the *GNAQ/11* mutant UM, respectively. Tumor heterogeneity of *GNAQ/11* mutations was also observed in a subset of tumors.

**Conclusions:**

Our results indicate that there is marked variation in MAPK activation in UM with *GNAQ/11* mutations. Thus, *GNAQ/11* mutational status is not a sufficient biomarker to adequately predict UM patient responses to single-agent selective MEK inhibitor therapy.

Uveal melanoma (UM) is the most common primary intraocular tumor in adults. Currently, there is no effective therapy for metastatic UM and research is ongoing to identify promising targeted therapies. Activation of mitogen-activated protein kinase (MAPK) has been observed in UM.^1^ It has been suggested that such activation is mostly due to somatic mutations in *GNAQ* and *GNA11* (*GNAQ/11*).^2^–^4^ Somatic mutations in *GNAQ/11* are reported in more than 80% of the UM tumors.^2^–^5^ With the exception of blue nevi and melanomas of the central nervous system, somatic mutations in *GNAQ*/*11* are unique to UM and have not been reported in other cancers.^6^ The mutations in both genes occur mostly in two loci one in exon 4 (codon 183) and the other in exon 5 (codon 209). Codon 209 mutations in both genes are much more common than codon 183 mutations.^4^ Mutations in another two genes *PLCB4* and *CYSLTR2* leading to constitutively activated G-protein signaling have also been reported in UM, although at much lower frequencies than *GNAQ/11*.^7^,^8^ Mutations in *GNAQ*, *GNA11*, *PLCB4*, and *CYSLTR2* are mostly mutually exclusive.^7^,^9^ The contribution of *PLCB4* and *CYSLTR2* mutations to MAPK activation is still not clear.

It has been suggested that selective inhibitors of MAPK pathway could be useful targets for therapy in patients with metastatic disease.^4^ This has been supported by in vitro studies and a phase II clinical trial of the selective MEK inhibitor selumetinib (AZD6244) for metastatic uveal melanoma^10^ where a modest improvement of progression-free but not overall survival was observed. However, such outcomes were not replicated in a phase III clinical trial of combined selumetinib and chemotherapeutic agent temozolomide.^11^ Interestingly, in both trials the response to therapy did not correlate with the *GNAQ/11* mutation status. It has been suggested that resistance genes such as the RNA helixase *DDX21* and the cyclin-dependent kinase regulator *CDK5R1* could play an important role in lack of response to selumetinib in *GNAQ/11* mutant UM.^12^ Intratumoral variation in the degree of MAPK activation could be additional explanation for the lack of response to selective MEK inhibition in a subset of tumors.^13^,^14^ A study on primary UM with *GNAQ*^Q209L/P^ mutation suggested that a subset of tumors with the mutation showed weak or no activation in MAPK.^15^ However, that report was limited by studying only *GNAQ* codon 209 mutations and the small number of samples included.^15^ Our current study was conceived prior to the two clinical trials and was carried out to validate our preliminary findings that there was a lack of MAPK activation in a significant number of UM primary tumors with somatic *GNAQ/11* mutations.^16^ In addition to confirming our earlier findings, we identified significant heterogeneity of MAPK activation within individual tumors. These findings provide an additional explanation for the lack of response to selective MEK inhibition in a subset of tumors and reveal the need to develop additional biomarkers to predict UM tumor responses to selective MEK inhibitors.

## Methods

### Patient Samples

A total of 42 primary UMs from patients treated with enucleation as primary therapy were included in our study. Archival materials were available for 39 of these tumors. Sufficient snap frozen tumor tissues were available for 17 tumors and both archival material and frozen tissues were available for 14 tumors. For three tumors, only snap frozen tissue was available. Control tissues for Western blot were obtained from the choroid of three (nontumour) eyes obtained from the Ohio's Lions Eye Bank and collected within less than 24 hours from the time of death. All specimens (UMs and normal controls) were collected per institutional ethical review board approved protocols (2003C0057 and 2006C0045) and in accordance with the tenets of the Declaration of Helsinki.

### Cell Lines

UM cell lines MEL202 (originally established by Bruce R. Ksander and Timothy G. Muray), and 92.1 (originally established by Martine J. Jager) were obtained from the European Searchable Tumour Cell Bank and Database. UM cell line MEL270 (originally established by Bruce R. Ksander and Timothy G. Muray) was provided by Martine J. Jager. Jager and colleagues have previously described the origin of these cell lines.^17^ Authentication of the cell lines was carried-out using short tandem repeat (STR) profiling.^18^,^19^ The cell lines were grown in RPMI media (Gibco, Carlsbad, CA, USA) supplemented with 10% heat-inactivated fetal bovine serum and 1% penicillin/streptomycin. Normal retinal pigmented epithelial cells (ARPE-19) were obtained from American Type Culture Collection (ATCC, Manassas, VA, USA) and grown according to the provider's protocol. UM7007 is a primary UM cell culture established in our laboratory. Both UM7007 and ARPE-19 were confirmed to have no *GNAQ/11*, mutations, whereas MEL202, 92.1 and MEL270 all were confirmed to have *GNAQ* codon 209 mutation.^20^

### DNA Extraction and Mutational Screening

DNA was extracted from archival material and/or fresh frozen tissue using a commercial extraction kit (DNeasy; Qiagen, Valencia, CA, USA), while microdissected tissue was extracted using a commercial kit (QIAamp DNA Microkit; Qiagen). Microdissection of tumor areas with differential staining for pERK1/2 was carried out from unstained sections adjacent to the stained tumor sections utilizing a surgical microscope and a 22G needle.

Mutational screening was carried out by restriction-fragment length polymorphism (RFLP). Supplementary Tables S1 and S2 summarize the primers and restriction enzymes used and the expected fragments sizes. All restriction enzymes were obtained from New England Biolabs (Ipswich, MA, USA). Direct (Sanger) sequencing was used for confirmation of mutations in *GNAQ/11* and mutational screening for hotspot mutations in *PLCB4* (codon 630) and *CYSLTR2* (codon 129). Sequencing was carried out at The Ohio State University Comprehensive Cancer Center Genomic Shared Resources utilizing a DNA sequencer (3730 DNA Analyzer; Applied Biosystems, Foster City, CA, USA). The sequence results were read by aligning with the reference sequence provided in Genebank accession numbers NM_002072.2 (*GNAQ*), NM_002067 (*GNA11*), NM_000933 (*PLCB4*), and NM_001308471 (*CYSLTR2*) utilizing the Sequencher software (version 4.8; Gene Codes Corp, Ann Arbor, MI, USA).

### *GNAQ* and *GNA11* Transient Transfection

Plasmids containing the *GNAQ* (wild type), *GNA11* (wild type), *GNAQ*^Q209L^, and *GNA11*^Q209L^ cDNAs were obtained from the Missouri S&T cDNA Resource Center. All the constructs contained an internal epitope tag (Glu-Glu; Sigma-Aldrich Corp., St. Louis, MO, USA) and were verified by sequencing. The primary uveal melanoma culture (UM7007) and the ARPE-19 cell line, both with no GNAQ/11 mutations, were used for transfection. Cells were grown in duplicates in six well plates to 75% to 80% confluence in RPMI medium containing 10% FBS, and 1% Penicillin/Streptomycin. Then they were transiently transfected with 4 μg of plasmid pcDNA3.1+ constructed with complete coding regions of wild type *GNAQ*, and *GNA11*, and mutant *GNAQ*^Q209L^, and *GNA11*^Q209L^ genes using a transfection reagent (Lipofectamine 2000; Invitrogen, Carlsbad, CA, USA). Cells transfected with the empty vector or treated with only lipofectamine 2000 were used as controls. Cells were grown in medium (Opti-MEM I Reduced Serum Medium, Cat. No. 31985-062; Invitrogen) and lysed 24 hours posttransfection.

### Immunohistochemistry and Western Blot

The activation of MAPK and AKT pathways was assessed by Western blot for the levels of phosphorylated ERK1/2, MEK1/2, and AKT proteins. Activation of MAPK in tumors was also assessed by immunohistochemistry using antibody for pERK1/2. Immunohistochemistry was carried-out on 39 formalin-fixed paraffin embedded primary UMs and Western blot was carried-out on 17 fresh frozen tumor specimens. For 14 tumors, both Western blot and immunohistochemistry were performed (see Supplementary Table S3). Antibodies for phospho p44/42 MAPK (pERK1/2-Thr202/Tyr204), phospho MEK1/2 (pMEK1/2-Ser217/221), and phospho AKT (pAKT-Ser473) were obtained from Cell Signaling Technology (Danvers, MA, USA) and β-Actin antibody was obtained from Sigma-Aldrich Corp.

For Western blot analysis, total proteins were extracted from tumor tissues and cell lines by incubation for 10 minutes with ice cold 1X cell lysis buffer (Cell Signaling Technology) spiked with 1 mM PMSF and 1X phosphatase inhibitor cocktail 2 (Sigma-Aldrich Corp.) immediately before use. The protein concentration was determined using a BCA protein assay kit (Pierce Biotechnology, Rockford, IL, USA). For Western blot analysis, 10 to 30 μg of protein extracts/sample were loaded on 12% Tris-HCl gel and transferred to Trans-Blot nitrocellulose filters (Bio-Rad Laboratories, Hercules, CA, USA). The primary and secondary antibodies were used at 1:1000 and 1:2000 dilutions respectively. Signals were developed using a chemiluminescent substrate (Super Signal West Pico Chemiluminescent Substrate; Pierce Biotechnology, Rockford, IL, USA). Densitometry was carried out on the scanned Western blot images using image analysis (Alphaease FC, software version 6.0.0; Alpha Innotech Corp., San Leandro, CA, USA) Data are representative of two experiments.

For immunohistochemistry, deparaffinized tissue sections were heat pretreated with citrate buffer, pH of 6, and incubated overnight at 4°C with pERK1/2 antibody at a 1:200 dilution. Immunostaining omitting the primary antibody was used as negative control. For detection of the immunostaining, chromogen (Vector NovaRED; Vector Laboratories, Burlingame, CA, USA) was utilized to produce a dark red staining to minimize the interference from melanin pigment. After counterstaining with hematoxylin and mounting, the slides were evaluated under a light microscope by two independent investigators (MHA, CMC) masked in regard to the mutation status of each tumor. Based on the correlation between immunostaining and Western blot analysis in the 14 tumors, staining limited to ≤1% of the tumor was considered negative (pERK1/2 score = 0), staining >1% to <10% was considered weak (score = 1), and staining ≥10% of the tumor was considered moderate to strong (score = 2). Staining of retina was utilized as an internal positive control for assessment of the adequacy of the immunostaining assay. Tumor areas with intense melanin pigmentation were excluded from our analysis.

### Statistical Analysis

Pearson's *χ*^2^ test was used to assess the association between ERK1/2 activation and *GNAQ/11* mutation status.

### Analysis of the Cancer Genome Atlas (TCGA) Data

Variant call files (VCF) of reported somatic mutations in uveal melanoma were downloaded from the TCGA project website. Allele frequencies of mutations in *GNAQ*/*11* were extracted using the query function within BCF tools of SAM (Sequence Alignment/Map) format and tools.^21^

## Results

### Frequency of *GNAQ*/*11* Mutations in Uveal Melanoma

The Table summarizes the frequency of *GNAQ/11* mutations observed in primary UM included in our study. Out of the 42 primary UM evaluated, 34 (80.9%) had mutations in only one of the *GNAQ/11* genes. One (2.3%) UM tumor had mutations in both genes and one tumor had two different mutations in *GNA11*, while seven (16.7%) were *GNAQ/11* wild type. Most mutations (30/35) were in codon 209 of *GNAQ*/*11*. All codon 209 *GNA11* mutations were A>T (*GNA11*^Q209L^). Seven of the *GNAQ* mutations were A>T *GNAQ*^Q209L^ and six were A>C *GNAQ*^Q209P^.

**Table:** Summary of GNAQ and GNA11 Mutations in Samples Included in the Study

**Status**	***n***	**%**
*GNAQ*^R183^	3	7.1
*GNAQ*^Q209^	12	28.6
*GNAQ*^Q209^ + *GNA11*^Q209^	1	2.4
*GNA11*^R183^	2	4.8
*GNA11*^Q209^	16	38.1
*GNA11*^R183^ + *GNA11*^Q209^	1	2.4
Total *GNAQ* or *GNA11* mutant	35	83.3
Total with no *GNAQ*/*GNA11* mutation	7	16.7
Total tumors	42	100

Of the three UM cell lines included in the study, two cell lines (92.1 and MEL202) had a heterozygous *GNAQ*^Q209L^ mutation, whereas MEL270 showed a homozygous *GNAQ*^Q209P^ mutation. The primary UM cells UM7007 and the retinal pigment epithelial cell line ARPE-19 were negative for *GNAQ* and *BRAF*^V600E^ mutations. None of the cell lines studied had a mutation in *GNA11*.

### Variability in ERK1/2 and MEK1/2 Activation in Uveal Melanoma

**Figure 1:**
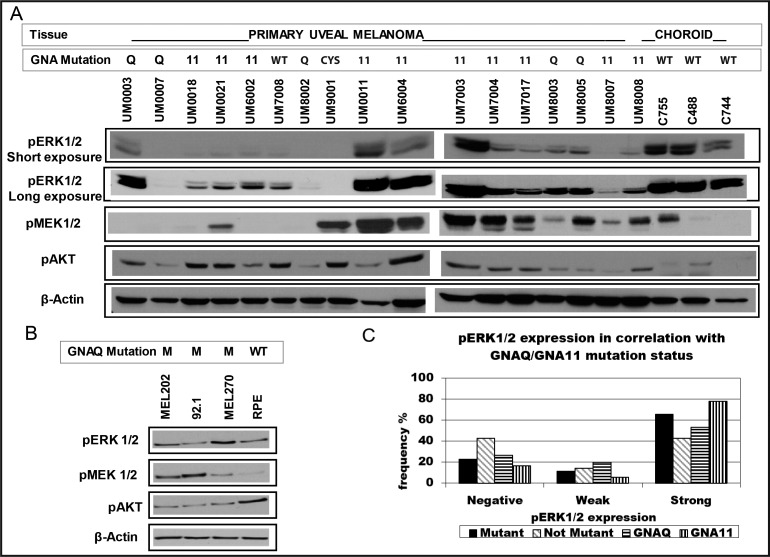
Expression of downstream targets in UM. (A) Western blot analysis of 17 primary UM and three nontumor choroids. Variations in pERK1/2 and pMEK1/2 levels were observed in tumors with GNAQ/11 mutations. Most tumors showed lower levels than normal choroidal samples suggesting a lower activation. Almost all GNAQ/11 mutant tumors showed higher levels of pAKT than normal choroidal samples suggesting activation of PI3K/AKT pathway in most UM. (B) Baseline pERK1/2, pMEK1/2 and pAKT levels in different UM cell lines and in ARPE-19 cells. GNAQ mutant cell lines (92.1, MEL202 and MEL270) showed variation in pERK1/2 and pMEK levels. (C) pERK1/2 levels in 39 primary UM by immunohistochemistry. A subset of UM with GNAQ/11 mutation show weak or no pERK1/2 immunostaining.

Western blot was carried out on the 17 tumors with available fresh frozen tissue and frozen choroid from three normal cadaver eyes. Five of these 17 tumors had *GNAQ*^Q209^ mutation, 10 had *GNA11*^Q209^ mutation and two didn't have mutation in either gene. The three normal choroid samples showed strong pERK1/2 but variable pMEK1/2 and pAKT levels. Out of the 15 tumors with mutation in either *GNAQ/11*, 14 (93.3%) showed detectable but highly variable pERK1/2, 10 (66.7%) showed pMEK1/2 and 15 (100%) showed pAKT levels. The tumor with *CYSLTR2* mutation showed detectable pMEK1/2 but no pERK1/2. Six tumors with no detectable pMEK1/2 showed pERK1/2 suggesting that activation of ERK1/2 in UM could be independent of MEK1/2 activation. Using densitometry, the pERK1/2 levels in primary UM was weaker in 11/15 of the tumors compared to nontumor choroids from cadaver eyes. One tumor (UM9001) showed no expression of pERK1/2 by Western blot (Fig. 1A) and less than 1% of tumor cells showed positive staining, Supplementary Table S3. The pAKT level was higher in all tumors compared to the controls while pMEK1/2 was detected in 10 (66.7%) tumors. UM cell lines with *GNAQ*^Q209^ mutations (92.1, MEL202 and Mel270) showed variable pERK1/2 and pMEK1/2 levels, Figure 1B.

**Figure 2:**
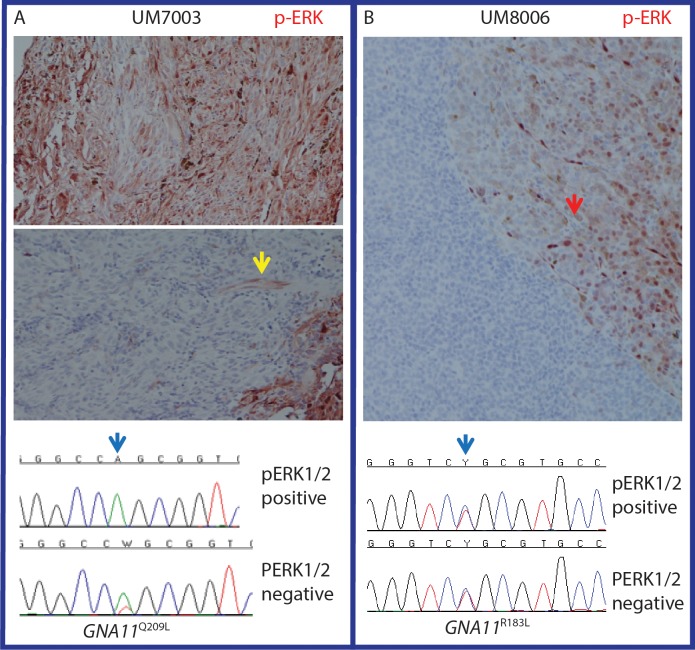
Tumor heterogeneity for MAPK activation may or may not associate with GNA11 mutational status. (A) Differential mutation status of a UM with high and no pERK1/2 immunostaining. Areas with strong staining showed no mutation while areas with no-staining showed heterozygous GNA11^Q209L^ mutation. Yellow arrow highlights a blood vessel with strong pERK1/2 staining in a negative staining part of the tumor. Blue arrow highlights the location of the mutation. Only tumor regions with negative pERK1/2 staining showed the mutation. (B) Example of a tumor with prominent heterogeneity of pERK1/2 that was not associated with mutation status of GNA11. The red arrow shows tumor area with strong pERK1/2 staining. The blue arrow highlights the location of the mutation. Both the pERK1/2 positive and negative parts of the tumor showed the mutation.

The activation of ERK1/2 was also assessed by immunohistochemistry using the same pERK/2 antibody in 39 primary tumors and correlated with *GNAQ/11* status (13 with *GNAQ* mutation, 18 with *GNA11* mutation, one with *GNAQ* and *GNA11* mutations, and seven with no mutation in both genes). In 14 of these UM, Western blot results were available. Both IHC and Western blot detected pERK1/2 in 14/15 tumors, Supplementary Table S3. Of the UM tumors with *GNAQ/11* mutation, 23/35 (65.7%) showed strong to moderate staining for pERK1/2 (in ≥10% of tumor cells) with only (28.6%) showing staining in >50% of tumor cells. Four tumors (11.4%) showed weak staining (in 1%–9%) and 8/35 (22.9%) showed no staining, Figure 1C. The staining of pERK1/2 was not uniform throughout the UM primary tumors, Figure 2. Of the seven tumors with wild type *GNAQ/11*, three tumors (42.9%) showed no staining, one tumor (14.3%) showed weak and three tumors (42.9%) showed strong uniform staining. No statistically significant difference was observed between tumors with and without *GNAQ/11* mutations (*P* = 0.49).

### *GNAQ* and *GNA11* Tumor Heterogeneity

Through reviewing the RFLP and sequencing results we observed variations in the mutant/wild type allele ratios in different tumors. Such variations strongly suggested tumor heterogeneity. To explore that possibility, we microdissected four tumors with large areas of significant differential pERK1/2 staining by immunohistochemistry, Figure 2. Two of these tumors had the *GNA11^Q209P^*, one had the *GNA11^R183C^* and one had the *GNAQ^Q209P^* mutations.

Variation in *GNA11* mutation status was observed in two out of the four tumors, Figure 2. In the remaining two tumors both the stained and unstained areas of the tumor showed heterozygous mutation. Interestingly, in the two tumors showing variation in *GNA11* mutation, the areas with strong pERK1/2 immunostaining showed no detectable *GNA11* mutation, while the tumor areas with no pERK1/2 immunostaining showed heterozygous *GNA11* mutation, Figure 2A.

### *GNAQ* and *GNA11* Allele Heterogeneity in a Subset of UM Primary Tumors From the TCGA Project

Out of the 80 tumors sequenced through the NIH TCGA (The Cancer Genome Atlas) project, 72 had a single mutation in either *GNAQ* or *GNA11* and two tumors had two mutations, one in each gene. The average allele frequency of mutations in *GNAQ* was 0.42 (range, 0.07–0.65) and of *GNA11* was 0.41 (range, 0.05–0.56). Four tumors had somatic mutation allele frequency less than 0.25 (0.24, 0.23, 0.07, 0.05). The two tumors with very low mutation allele frequencies (0.05 and 0.07) were those with mutations in both genes. This suggests that in a small subset of tumors the variation in MAPK activation could be due to allele heterogeneity.

### *GNAQ* and *GNA11* Transfection and Downstream Signaling in UM and Control Cell Lines

**Figure 3:**
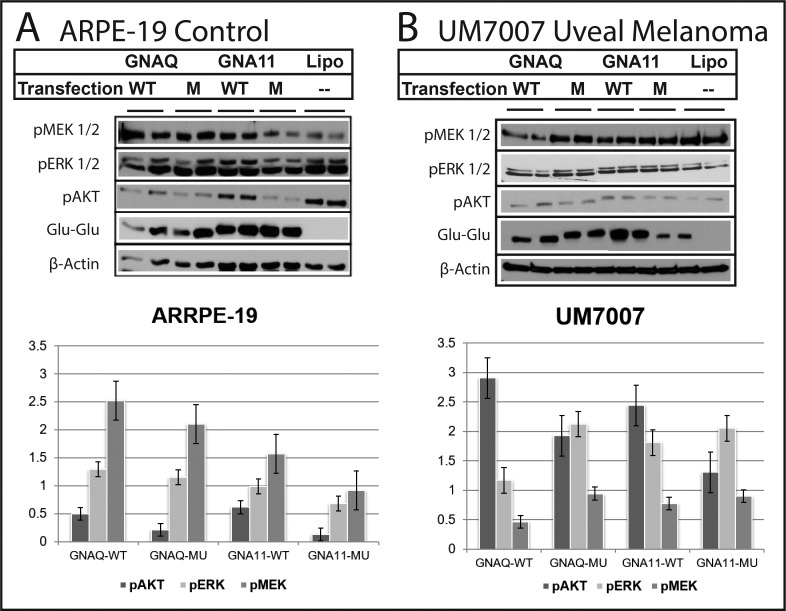
Differential activation of downstream signaling in cells transfected by wild type and mutant GNAQ/11. (A) UM7007 a primary culture from a UM tumor. Activation of ERK1/2 was more prominent in tumor cells transfected with mutant GNAQ/11 plasmids. Activation of AKT was more prominent in cells transfected with wild type GNAQ/11 plasmids. MEK1/2 was not activated. (B) In the RPE (ARRPE-19 cells) the ERK1/2 pathway was not significantly activated with both mutant and wild type GNAQ/11. Suppression of the AKT activation was prominent. Activation of the MEK1/2 was observed but more significantly in cells transfected with the wild type plasmids. Lipo, lipofectamine only.

To study the downstream signaling of *GNAQ/11* we selected two cell lines with no detectable mutation in these genes. Variations in downstream signaling between UM (UM7007) and control cell lines (ARPE-19) were observed after transfection of wild-type or mutant *GNAQ/11*, Figure 3. The efficiency of transfection was confirmed by the expression of epitope (Sigma-Aldrich Corp.) used to tag the transfection vector, Figure 3. Activation of ERK1/2 was observed in UM7007 cells transfected with both mutant and wild-type *GNAQ/11*, while ARPE-19 control cells did not show ERK1/2 activation with transfection of either constructs. On the other hand, activation of pMEK1/2 was observed in ARPE-19 cells but not the UM7007 transfected cells compared to the no transfection controls. Suppression of AKT activation was observed in the ARPE-19 cells in particular with the mutant constructs but not in UM7007 which showed activation of AKT. Our results indicate that the activation of downstream signaling in *GNAQ/11* transfected cell lines is dependent on the background of the cell line linage and likely other genetic alterations in the tumors.

## Discussion

It has been suggested that the oncogenic effect of somatic mutations in *GNAQ/11* in UM is mostly through activation of the MAPK pathway. Our study supports the reported high frequency of *GNAQ/11* mutations in primary UM tumors^2^,^4^ but also identifies that a significant subset (34.3%) of these primary UM tumors with *GNAQ/11* mutation have absent or low-level activation of the MAPK pathway with considerable tumor heterogeneity. Tumor areas with confirmed mutation in *GNAQ/11* showed variation in activation of the MAPK pathway suggesting that these mutations are not sufficient for MAPK activation in vivo. This lack of direct MAPK activation resulting from *GNAQ/11* mutation in UM was also evident in our transfection experiments that indicate that there may be cell-specific differences in the levels of MAPK activation due to in *GNAQ/11* mutation.

The low-level of MAPK activation in primary UM with *GNAQ* mutation has been reported by Populo et al.^15^ In that study using immunohistochemistry absent and weak levels of activated MAPK were observed in 2/8 and 3/8 of the tumors respectively.^15^ Khalili et al.^20^ showed that UM cell lines with mutation in *GNAQ/11* had lower baseline activation of MAPK compared to cell lines (MEL285 and MEL290) with wild-type *GNAQ/11*. Furthermore, in a transgenic zebrafish UM tumor model Mouti et al.^22^ reported weak pERK1/2 staining in established zebrafish UM tumors, which was in contrast to the strong pERK1/2 immunoreactivity in the oncogenic RAS-driven skin lesions that developed in the model. Also, no changes were observed in pERK1/2 levels upon transient knockdown of *GNAQ* in the Mel270, OMM1.3, OMM1.5 (GNAQ*^Q209P^*) and the MEL202 (GNAQ*^Q209L^*) mutant UM cell lines and only the 92.1 (GNAQ*^Q209L^*) showed significant inhibition.^22^ It has been suggested that episodic Src activation is associated with ERK1/2 activation in primary but not metastatic UM.^23^ Taken together, these studies indicate that the activation of the MAPK pathway is not a major downstream target in a subset of UM with *GNAQ/11* mutation.

Targeted therapy utilizing selective MEK inhibition appears promising for uveal melanoma based on preliminary results of a phase II clinical trial; however, a subset of patients with *GNAQ/11* mutation are resistant to this therapy.^12^,^24^ It has been suggested that the resistance is due to existence of a unique subset of “MEK-resistant genes” in a subset of *GNAQ* mutant tumors.^12^ Our findings provide another possible explanation and suggest that tumor heterogeneity and variability of MAPK activation could be an important cause for therapy resistance. We speculate that patients with *GNAQ/11* mutation who are resistant to selective MEK inhibitors have a greater proportion of tumor cells that lack MAPK activation. In vitro studies of UM cell lines with *GNAQ* mutations show weaker response to the B-Raf inhibitor PLX4720 and the MEK inhibitor selumetinib than *BRAF* mutant cells.^13^ Further studies are needed to correlate the degree of MAPK activation and the clinical response to selective MEK inhibitor therapy in metastatic UM. Given the potential clinical implications of a lack of correlation of *GNAQ/11* mutation with ERK1/2 activation, our study highlights the need to develop additional biomarkers to assay MAPK activity in the tumor, rather than *GNAQ/11* mutation status alone. This might better predict which patients may respond well to selective MEK inhibitor treatment.

In vitro studies have shown that combinations of MAPK and PI3K pathways inhibition are more effective in controlling *GNAQ* mutant UM^16^,^20^ and reducing the proliferation of UM cells.^14^ The high frequency of AKT activation identified in our study suggests that such an approach may have a beneficial effect as well in vivo.

It has been suggested that *GNAQ* mutation occurs early in the tumorigenesis of UM.^3^ This was based in large part on the lack of association between *GNAQ* and any clinical, pathologic, or molecular features associated with late-tumor progression and the identification of these mutations in uveal nevi.^4^,^25^ Since initiating tumor mutations should be observed as a clonal genetic alteration in all tumor cells, our study suggest that although *GNAQ/11* mutations occur early in tumor development in the majority of UM tumors, a small subset of patients develop these mutations as later events in tumor progression.

The lack of association between the *GNAQ/11* mutations and any clinical, pathologic, or molecular prognostic features of UM is likely because of the high frequency of these mutations in UM.^5^,^26^ In addition, the variation in the downstream signaling of wild-type and mutant *GNAQ/11* in different cell lines suggests that the downstream molecular effects depend on other genetic alterations in the tumors. Future studies are needed to characterize the modifiers of G-protein–MAPK pathway interactions in UM.

Our study focused on *GNAQ/11* mutations; however, mutations in two additional genes *PLCB4* and *CYSLTR2* have been reported as additional G-protein signaling activators.^7^,^8^ The impact of mutations in *PLCB4* and *CYSLTR2* on MAPK pathway activation has not been reported. In our cohort we identified one tumor with *CYSLTR2* mutation. The tumor showed activation of MEK1/2 and AKT but not ERK1/2. Further studies of the effect of *PLCB4* and *CYSLTR2* on MAPK and AKT pathway activation are warranted.

In conclusion, our study indicates that primary UM tumors have heterogeneity in MAPK activation and *GNAQ/11* mutation. In contrast to the dogma that *GNAQ/11* mutation leads directly to MAPK activation and tumor survival, there is a subset of *GNAQ/11* mutated UM tumors which lack significant MAPK activity. This finding suggests that *GNAQ/11* mutational status alone may not be a sufficient biomarker to adequately predict UM patient responses to single-agent selective MEK inhibitor therapy for UM and that additional biomarkers, such as direct measurement of UM MAPK activation need to be developed. The lack of MAPK activation in a subset of *GNAQ/11* mutated UM should also be explored as a potential major resistance factor to targeted therapy.

## Supplementary Material

Supplement 1

Supplement 2

Supplement 3
