# Supplementary material for: Heterogeneity in Mitogen-Activated Protein Kinase (MAPK) Pathway Activation in Uveal Melanoma With Somatic GNAQ and GNA11 Mutations
Source: Invest Ophthalmol Vis Sci. 2019 Jun;60(7):2474–80. doi: 10.1167/iovs.18-26452 (PMC6557618; doi:10.1167/iovs.18-26452)
Supplement: Supplement 1 [file iovs-60-06-18_s01.pdf]

Supplement Table 1. PCR primers used for restriction fragment polymorphism and sequencing

Gene	Exon	Codon	Transcript	Forward Primer 5'→3'	Reverse Primer 5'→3'	Product size (bp)
GNAQ	4	183	NM_002072.2	TGGTGTGATGGTGTCACTGACATTCTCAT	AGCTGGGAAATAGGTTTCATGGACTCAGT	212
GNAQ	5	209	NM_002072.2	TTTTCCCTAAGTTTGTAAGTAGTGC	CCCACACCCTACTTTCTATCATTTAC	298
GNA11	4	183	NM_002067	GTGCTGTGTCCCTGTCCTG	GGCAAATGAGCCTCTCAGTG	249
GNA11	5	209	NM_002067	GGTGGGAGCCGTCCTGGGAT	GGCAGAGGGAATCAGAGGGGC	344
PLCB4	20	630	NM_000933	TGAGTCGCATTTACCCCAAG	AACTGATGGGAAGTGCTGGC	184
CYSLTR2	1	129	NM_001308471	TCATAAGCACGCTTCCCTTCA	ATTGCCAGGAAACGCACAAC	160
