# Supplementary material for: Heterogeneity in Mitogen-Activated Protein Kinase (MAPK) Pathway Activation in Uveal Melanoma With Somatic GNAQ and GNA11 Mutations
Source: Invest Ophthalmol Vis Sci. 2019 Jun;60(7):2474–80. doi: 10.1167/iovs.18-26452 (PMC6557618; doi:10.1167/iovs.18-26452)
Supplement: Supplement 2 [file iovs-60-06-18_s02.pdf]

Supplement Table 2. Enzymes and product sizes for restriction fragment length polymorphism assay

Gene	Enzyme	PCR product	RFLP band size in base pairs		
			WT	Heterozygous Mutation	Homozygous Mutation
$GNAQ^{R183}$	Taq I	212	141, 71, 23	141, 118, 71, 23	118, 71, 23
$GNAQ^{Q209}$	Eco0109I	298	298	292, 191, 107	191, 107
$GNA11^{R183}$	BstUI	249	139, 110	249, 139, 110	249
$GNAQ^{Q209}$	MspA1I	344	195, 98, 51	293, 195, 98, 51	293, 51

RFLP: Restriction Fragment Length Polymorphism
