# Supplementary material for: Heterogeneity in Mitogen-Activated Protein Kinase (MAPK) Pathway Activation in Uveal Melanoma With Somatic GNAQ and GNA11 Mutations
Source: Invest Ophthalmol Vis Sci. 2019 Jun;60(7):2474–80. doi: 10.1167/iovs.18-26452 (PMC6557618; doi:10.1167/iovs.18-26452)
Supplement: Supplement 3 [file iovs-60-06-18_s03.pdf]

Supplement Table 3 Correlation between immunohistochemistry and Western blot analysis

| **Sample** | **Tumor MHA** | **%** | **Tumor CMC** | **%** | Average IHC | Western pERK |
|---|---|---|---|---|---|---|
| UM9001 | 2 | 1 | 3 | <1 | 0 | 0 |
| UM8005 | 2 | 10 | 3 | <5 | 2 | 2 |
| UM0021 | 1 | 10 | 2 | 15 | 2 | 2 |
| UM7008 | 2 | 100 | 3 | >90 | 2 | 2 |
| UM6004 | 2 | 10 | 2-3 | 10 | 2 | 3 |
| UM7003 | 2 | 10 | 3 | 10 | 2 | 3 |
| UM8008 | 2 | 10 | 2 & 4 | <10 & <5 | 2 | 1 |
| UM8007 | 1 | 20 | 2 | 20 | 2 | 1 |
| UM8003 | 2 | 30 | 2 | 70 | 2 | 2 |
| UM0011 | 2 | 35 | 2, 3 | 10%, 20% | 2 | 3 |
| UM7004 | 2 | 40 | 3 | 90 | 2 | 2 |
| UM8002 | 2 | 50 | 2-4 | 60 | 2 | 1 |
| UM7017 | 2 | 40-50 | 2 | 60 | 2 | 2 |
| UM6002 | 2 | <5 | 1 | 5 | 1 | 2 |
